# Bias-Enhanced Formation of Metastable and Multiphase Boron Nitride Coating in Microwave Plasma Chemical Vapor Deposition

**DOI:** 10.3390/ma14237167

**Published:** 2021-11-25

**Authors:** Kallol Chakrabarty, Paul A. Baker, Vineeth M. Vijayan, Shane A. Catledge

**Affiliations:** Department of Physics, University of Alabama at Birmingham, Birmingham, AL 35294, USA; kallol89@uab.edu (K.C.); pabaker@uab.edu (P.A.B.); vineeth@uab.edu (V.M.V.)

**Keywords:** ceramics/coating materials, chemical synthesis, vapor deposition, mechanical properties

## Abstract

Boron nitride (BN) is primarily a synthetically produced advanced ceramic material. It is isoelectronic to carbon and, like carbon, can exist as several polymorphic modifications. Microwave plasma chemical vapor deposition (MPCVD) of metastable wurtzite boron nitride is reported for the first time and found to be facilitated by the application of direct current (DC) bias to the substrate. The applied negative DC bias was found to yield a higher content of *sp*^3^ bonded BN in both cubic and metastable wurtzite structural forms. This is confirmed by X-ray photoelectron spectroscopy (XPS) and Fourier transform infrared spectroscopy (FTIR). Nano-indentation measurements reveal an average coating hardness of 25 GPa with some measurements as high as 31 GPa, consistent with a substantial fraction of *sp*^3^ bonding mixed with the hexagonal *sp*^2^ bonded BN phase.

## 1. Introduction

Boron nitride (BN) is a covalently bonded ceramic material and a member of the Group III nitrides. It exhibits many outstanding properties, such as chemical inertness, thermal conductivity, very good mechanical properties, and electrical insulating properties [1,2,3]. Boron nitride (BN) exists in multiple forms, including amorphous BN (*a*-BN), hexagonal BN (*h*-BN), rhombohedral BN (*r*-BN), cubic BN (*c*-BN), and wurtzite BN (*w*-BN). While *r*-BN is basically *h*-BN with a slight variation in the stacking of individual layers, *w*-BN is distorted *c*-BN. Both *r*-BN and *h*-BN have *sp*^2^ hybridization, while *c*-BN and *w*-BN have *sp*^3^ hybridization in their covalent bond, thus giving rise to widely varying properties of the material. The hardness of cubic boron nitride is second only to diamond, but *c*-BN has better oxidation resistance at high temperatures. Wurtzite boron nitride has a hardness which even exceeds diamond and can usually only be produced via shock compression methods, such as detonation, or static compression at high pressures [4]. The oxidization temperature of the *c*-BN and w-BN is also about twice the temperature of diamond in ambient environments and, unlike diamond, *c*-BN does not react with molten ferrous materials [5,6,7,8,9]. Among all the predominantly covalently bonded materials, *c*-BN and *w*-BN also have the widest band gap (6.2 ± 0.2 eV) and can be doped for both p-type and n-type conductivity [5,6,7,8,9,10,11,12,13]. Another interesting property of *c*-BN is its transparency to a broad range of electromagnetic radiation from the ultraviolet to the infrared [14]. The above-mentioned outstanding properties of *c*-BN and *w*-BN make it a very promising candidate for hard coatings in grinding and cutting tools, electronic and optoelectronic device applications such as deep-ultraviolet detectors, light-emitting diodes, high-power/high-speed transistors for operation at high temperatures in harsh environments, and UV–Vis–IR transparent optical devices such as lenses and windows [3,14,15,16]. It has recently been reported that *c*-BN can be biocompatible and non-cytotoxic, and can be used as a novel scaffold for biomedical applications [15].

Boron nitride is not found naturally; it has to be produced from boron and nitrogen precursors. Different methods and experimental conditions have been used to synthesize BN from a variety of boron and nitrogen precursors [17,18,19,20,21,22,23,24]. Preparation of boron nitride (BN) was reported from high pressure/high temperature synthesis using diamond anvil cell/laser heating. However, high pressure/high temperature synthesis can be expensive and often only generates very little amounts of material. In contrast, chemical vapor deposition (CVD) has proven to be a scalable technology for synthesizing a wide range of coating materials including BN coatings with large area uniformity and at the same time it is also very cost effective [17,18,19,20,24,25]. Laser chemical vapor deposition was also used to high yield synthesis of cubic and hexagonal boron nitride [26]. At ordinary temperatures and pressures *h*-BN is the stable structure of BN and *c*-BN at high temperatures and pressures. The wurtzite phase of BN is metastable at all pressures and temperatures and its stabilization is challenging because of the very low kinetic barrier for the transformation back to ambient phases [4,23]. Among the different allotropes of boron nitride, *c*-BN and *w*-BN are superhard in nature [27]. The *h*-BN phase is the most stable and therefore more easily formed under a wider range of conditions compared to the *c*-BN and w-BN phases [28]. *c*-BN has been deposited both by the low-pressure CVD method and by the high-pressure–high temperature process. Synthesis of the metastable wurtzite BN polymorph has only been reported by high-pressure–high temperature and shock compression methods [4,27,29,30,31]. Ion bombardment has been reported to be necessary in order to obtain *c*-BN coatings grown by CVD [32]. The objective of this research is to investigate deposition conditions using substrate bias leading to BN coatings containing a significant fraction of *sp*^3^ bonds. This is expected to yield much higher hardness than that offered by *h*-BN. In this work, we report the first evidence of metastable *w*-BN grown in a low-pressure plasma environment, facilitated by application of substrate bias. The *w*-BN is shown to form alongside *c*-BN and *h*-BN in this process.

## 2. Materials and Methods

### 2.1. MPCVD Process

Boron nitride coatings were grown in a microwave plasma chemical vapor deposition (MPCVD) system—details provided elsewhere [33,34]. Inside the MPCVD chamber, the substrate was heated by direct contact with the plasma. Water was used to cool both the sample stage and the outer resonance cavity jacket. From the resonance cavity a low-pressure plasma environment was isolated by a quartz bell jar. N-type (100)-oriented silicon substrates with 525 μm thickness were placed on the surface of a 0.5” diameter molybdenum screw located along the central axis of the bell jar. The substrate was cleaned in acetone, methanol, and then distilled water. Microwave power of 1 kW and chamber pressure of 15 Torr were used in the growth process. The carrier gas was hydrogen (H_2_) and in this study the reactive gas was a diborane mixture (95% H_2_, 5% B_2_H_6_) and N_2_. The gas flow rates were: 500 standard cubic centimeters per minute (SCCM) of hydrogen, 9 SCCM of the diborane mixture, and 0.4 SCCM of N_2_. Two types of samples were grown with the same experimental conditions at an average substrate temperature of 800 °C with the only variation being that one sample was synthesized without the presence of bias and the other was synthesized with the applied substrate bias. A Sorensen DCS 600-1.7E power supply (AMETEK Programmable Power, Inc. San Diego, CA, USA) provided the bias voltage to negatively charge the substrate while maintaining the chamber wall of the MPCVD grounded. The corresponding electric field that is created accelerates positively charged ions towards the substrate. Negative 400 V was used in the experiment resulting in an initial sample current of 20 mA. Maximum current was found using −400 V so in this study it was used as external voltage. The current decreased gradually to zero during the initial 15 min of deposition. This is believed to be due to the insulating nature of boron nitride that gradually forms on the silicon substrates. The external power supply was turned off after the current reached zero.

### 2.2. Characterization Techniques

Samples were examined using Fourier transform infrared spectroscopy (FTIR), X-ray photoelectron spectroscopy (XPS), glancing-angle X-ray diffraction (XRD), nano-indentation, and scanning electron microscopy (SEM). To learn the chemical properties of the coatings, the Bruker alpha FTIR spectrometer (Bruker Corporation, Billerica, MA, USA) was used (ranging from 1800 to 600 cm^−1^). The FTIR spectra were recorded using a total number of 1024 scans with a 4 cm^−1^ resolution. To determine the elemental compositions and chemical bonding of the boron nitride coating, XPS was carried out with a Phi Electronics Versaprobe 5000 (Phi Electronics, Chanhassen, MN, USA), equipped with a micro-focused Al monochromatic source (λ = 1486.6 eV). A Panalytical Empyrean X-ray diffractometer (Malvern, Panalytical, Almelo, Nederland) (Copper K_α__1_, λ = 1.54059 Å) was used to obtain the XRD pattern. A glancing-angle 2-theta scan with an angle of incidence of 1 degree was carried out to attain the XRD patterns. A hybrid monochromator (Malvern, Panalytical, Almelo, Nederland) with a 1/8° divergence slit and a 1/16° anti-scattering slit, as well as a parallel plate collimator (Malvern, Panalytical, Almelo, Nederland) on the diffracted beam path with a proportional detector (Malvern, Panalytical, Almelo, Nederland) were used in the XRD instrumentation. The nano-indentation hardness was measured using an Agilent Nano Indenter G200 (MTS Nano Instruments, Oak Ridge, TN, USA) with a Berkovich diamond tip with nominal radius of 50 nm. Before and after indenting our sample for calibration, a fused silica reference with an accepted Young’s modulus value of 72 GPa was evaluated. We can establish that the tip shape did not change during the examination of the sample because the range of Young’s modulus of this silica standard was consistent with the accepted value (before and after indenting our sample). All indents were created to a maximum depth of 600 nm, including those on the silica. The measured hardness was determined at maximum load. The morphology of the bias-enhanced coating was studied using the SEM instrumentation of an FEI Quanta TM 650 FEG Scanning Electron Microscope (Thermo Fisher Scientific, Hillsboro, OR, USA) at a 5 kV beam voltage.

## 3. Results

### 3.1. Fourier Transform Infrared Spectroscopy (FTIR)

**Figure 1:**
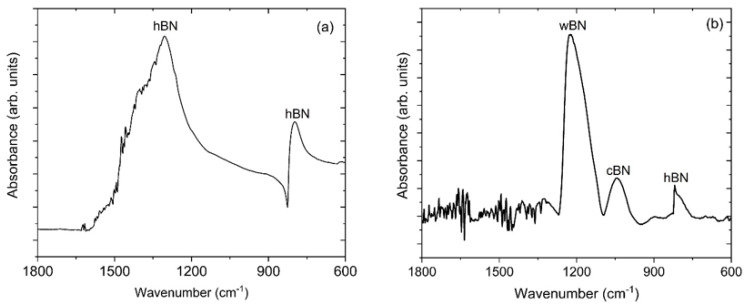
Fourier transform infrared spectroscopy (FTIR) of boron nitride coating (**a**) without applied bias and (**b**) with applied substrate bias. The coating grown without applied bias only shows *sp*^2^ (*h*-BN) bonded BN bonding, whereas the coating grown with applied bias shows a mixture of *sp*^2^ (*h*-BN) and *sp*^3^ (*c*-BN and *w*-BN) bonded BN.

FTIR spectral analysis was utilized to get information on the effect of biasing on the bonding environment of boron nitride. Figure 1 shows FTIR of the BN coatings grown (a) without applied bias and (b) with applied substrate bias. Both of the FTIR spectra exhibit several peaks in the fingerprint region (1500–500 cm^−1^). The BN coating deposited without a negative bias voltage shows two bands, at 1307 and 786 cm^−1^. These bands represent the in-plane (B-N) stretching mode and out-of-plane (B-N-B) bending modes of the *sp*^2^ bonded hexagonal (*h*-BN) phase, respectively [35,36]. These data show that the BN coating grown without applied bias consists primarily of the *sp*^2^ bonded hexagonal BN phase. In contrast, the BN coating grown with applied bias shows two additional peaks at 1045 and 1224 cm^−1^, corresponding to the Restrahlen band of *c*-BN and the TO modes of *w*-BN, respectively [35,37,38]. These observations clearly suggest that applied biasing introduces *sp*^3^ bonded cubic and wurtzite phases of BN. Taken together, the BN coating prepared without the applied bias consists of only the hexagonal phase whereas the BN coating prepared with the applied bias consists of a mixture of cubic, wurtzite and hexagonal phases. For the bias-enhanced BN coating, the wurtzite and cubic phases are more predominant compared to the hexagonal BN phase. The complete peak assignment is also given in Table 1. The volume fraction of the different BN phases of the bias-enhanced coating can be estimated by the relative intensities of the *w*-BN, *c*-BN and *h*-BN peaks using the following equation [39,40].

(1) $$Q_{w-\mathrm{BN}}=\frac{I_{w-\mathrm{BN}}}{I_{c-\mathrm{BN}}+I_{w-\mathrm{BN}}+I_{h-\mathrm{BN}}}$$

Using the above equation, we can calculate the relative content of *w*-BN of bias-enhanced BN coating. The estimated volume fraction of *w*-BN is 72% in the bias-enhanced BN coating. Using a similar kind of equation, we can estimate the volume fraction of *c*-BN and *h*-BN; the respective volume fractions of BN of *c*-BN and *h*-BN are 15% and 13%.

### 3.2. X-ray Photoelectron Spectroscopy

**Figure 2:**
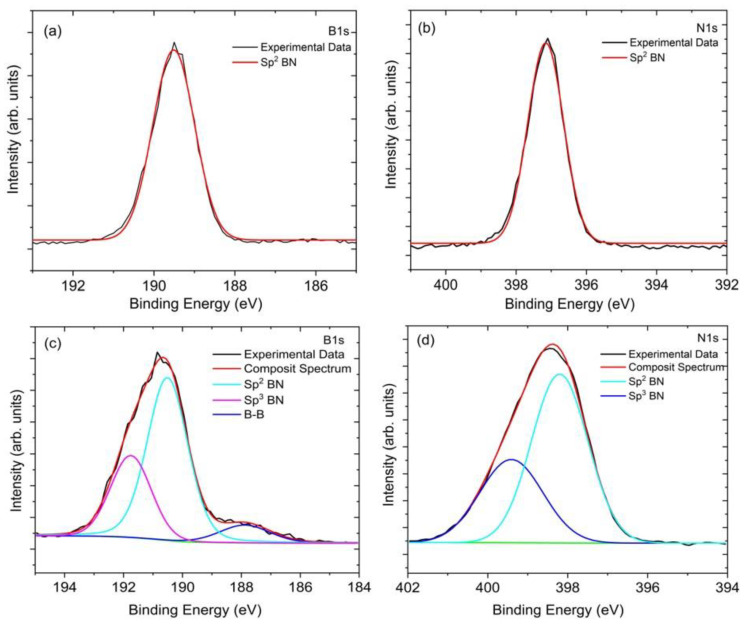
X-ray photoelectron spectra of hi-res (**a**) B1s and (**b**) N1s of without external bias BN coating shows only *sp*^2^ bonded BN. Hi-res (**c**) B1s and (**d**) N1s of bias-enhanced BN coating shows both *sp*^2^ and *sp*^3^ bonded BN on the spectrum. The detailed compositional analysis with corresponding binding energy is shown in Table 2.

X-ray photoelectron spectroscopy was carried out to determine the elemental compositions and chemical bonding of the boron nitride (BN) coatings. Figure 2a,b shows the high-res x-ray photoelectron spectra of B1s and N1s for BN coatings grown without applied bias. The binding energy peak positions for the hi-res B1s and N1s reveal fits to only *sp*^2^ bonded BN (ca. 189.7 eV and 397.2 eV, respectively) [42,43,44,45]. Figure 2c,d shows high-res x-ray photoelectron spectra of B1s and N1s for BN coatings grown with applied bias. These coatings reveal fits to both *sp*^2^ and *sp*^3^ bonded BN with the *sp*^3^ component ca. 191.8 eV and 399.5 eV for B1s and N1s, respectively [42,43,44,45]. From the survey spectra, the surface of the bias-enhanced BN coating was composed of 49.1% boron, 44.1% nitrogen, 4% carbon, and 2.8% oxygen (rel. at %) with no other elements present. The atomic ratio of boron and nitrogen was 1.1 and very close to the chemical stoichiometry of BN. A small amount of surface contamination due to adventitious carbon and oxygen is generally present in samples that have been exposed to air. The high-res B1s scan in Figure 2c shows that 8% boron is B–B bonded, 62% is *sp*^2^ BN bonded and 30% is *sp*^3^ BN bonded. The high-res N1s scan in Figure 2d shows 65% is *sp*^2^ BN bonded and 35% is *sp*^3^ BN bonded. The complete peak assignment is shown in Table 2.

### 3.3. X-ray Diffraction

**Figure 3:**
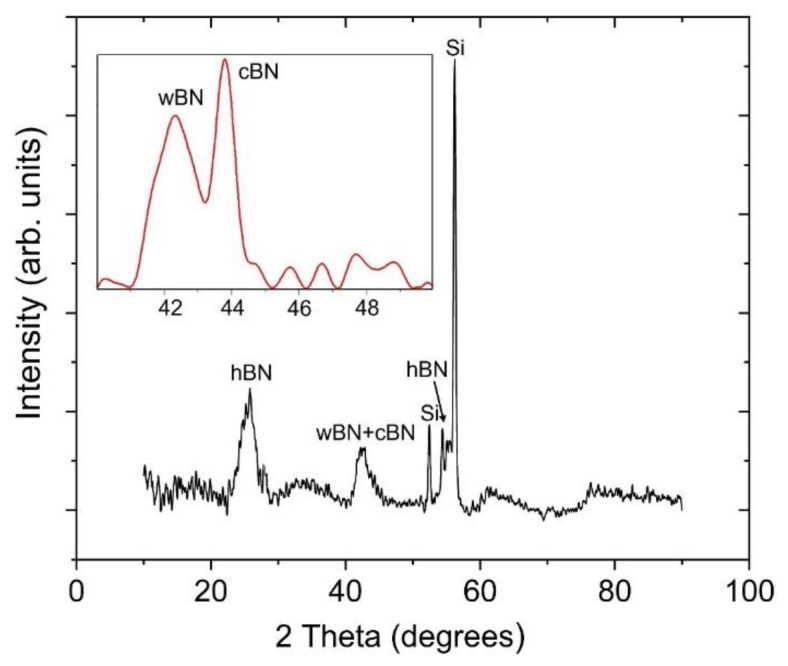
Glancing-angle X-ray diffraction pattern for bias-enhanced boron nitride coating. X-ray diffraction pattern of the coating grown without applied bias is reported in our previous work [28].

The results of the bias-enhanced boron nitride coating using a glancing-angle x-ray diffraction pattern are shown in Figure 3. Two broad diffraction peaks at 2θ of 24–28° and 41–45°, along with three other peaks at 52°, 55°, and 56° are present in the diffractogram. Sharp peaks near 52° and 56° may be seen on the single crystal Si substrates, which are often influenced by sample orientation during the XRD scan [28]. The peak around 26° in the XRD pattern is associated with the (002) reflection of *h*-BN. The other weak characteristic peak (004) of hexagonal boron nitride around 55° is also present [28,47,48]. The broad peak around 41–45° is the combination of *sp*^3^ bonded *w*-BN and *c*-BN. To get better visualization, high resolution XRD was performed at 2θ of 40–50°. The broad peak at 41–45° was resolved into two peaks which corresponds to *w*-BN (002) and *c*-BN (111) [15,42,49,50,51].

### 3.4. Nano-Indentation

**Figure 4:**
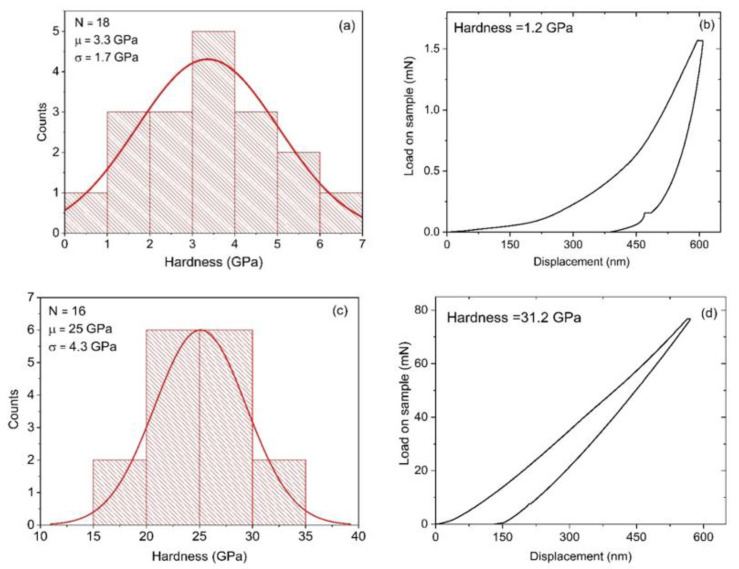
(**a**) Histogram from 16 indents showing hardness values for without-bias boron nitride coating. (**b**) Nanoindentation load/displacement curve from one location on the without-bias boron nitride coating at a depth of 600 nm. (**c**) Histogram from 16 indents showing hardness values for bias-enhanced boron nitride coating. (**d**) Nanoindentation load/displacement curve from one location on the bias-enhanced boron nitride coating at a depth of 600 nm. The extracted value of nano-indentation hardness 31.2 GPa is also indicated.

Figure 4 shows the nano-indentation hardness data of the boron nitride coatings. The indents were performed to a depth of 600 nm at several locations (n = 18). Figure 4a shows an average measured hardness of 3.4 ± 1.7 GPa for the coating grown without applied bias. The load/displacement curve of one representative indent is also shown in Figure 4b. The relative contribution of elastic and plastic deformation can be calculated from the final unloading depth of the load/displacement curve indicating a high plastic deformation of 67%. Figure 4c shows an average measured hardness (n = 16) of 25.0 ± 4.3 GPa for the coating grown with applied bias. Figure 4d shows the load/displacement curve of the indent with the highest recorded hardness of 31.2 GPa. The final unloading depth of the load/displacement curve can be used to calculate the relative contribution of elastic and plastic deformation. A high elastic recovery of 75% was found for the boron nitride coating grown with applied bias. Therefore, while the bias-enhanced coating has high elastic recovery, the coating grown without bias shows much higher plastic deformation.

### 3.5. Scanning Electron Microscopy (SEM)

**Figure 5:**
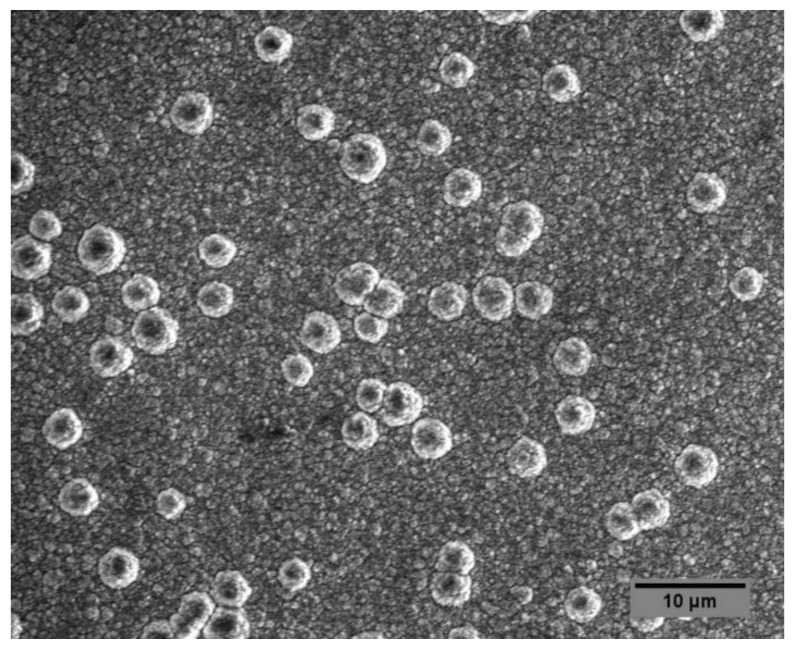
Scanning electron microscopy (SEM) image of bias-enhanced boron nitride coating. Morphology of the coating grown without applied bias is reported in our previous work [28].

The surface morphology of boron nitride coatings was evaluated using scanning electron microscopy, as shown in Figure 5. As reported in our previous study [28], the morphology of hexagonal boron nitride grown without applied bias shows plate-like structures which have been reported for nanocrystalline *h*-BN [52,53]. In contrast, the bias-enhanced BN surface shows rounded nodules ~5μm diameter protruding from a very fine-grained base structure.

## 4. Discussion

The challenge of bias-enhanced formation of boron nitride in MPCVD is in finding the range of bias voltage where *sp*^2^ bonds can transform to *sp*^3^ bonds. To the best of our knowledge, this work represents the first report of metastable wurtzite BN growth from a low-pressure plasma CVD process. Its formation is facilitated by application of substrate bias. Despite the low expected kinetic barrier for transformation to the more stable hexagonal phase, the wurtzite phase can be quenched and remain stabilized at ambient conditions, albeit in a mixture with hexagonal and cubic forms. According to the XPS and FTIR characterization, the sample prepared without applied bias shows only *sp*^2^ bonded BN. Application of substrate bias facilitates *sp*^3^ bonded BN, also confirmed by XPS, XRD and FTIR analysis. This is supported by the substantial increase in average coating hardness from 3.4 GPa to 25.0 GPa.

Possible mechanisms by which an increase in *sp*^3^ bonding occurs as well as in the stabilization of the thermodynamically metastable wurtzite BN phase involve the influence of ion bombardment-induced stress as well as crystalline defect-induced stabilization of the wurtzite phase. The formation of an electric field by applying a negative DC substrate bias directs the transport of positive ions towards the silicon substrate. These ions can impact with considerable kinetic energy to vibrationally excite target B and N atoms on the surface, leading to a local rearrangement of atomic positions and bonds. It is expected that the additional ion bombardment raises the atom mobility on the surface and boosts the displacement of the surface atoms. Highly energetic ions can be incorporated into the subsurface region of the substrate and increase compressive stress [32]. Stress is related to the ion energy and ion to atom arrival ratio, which can be described by ion momentum transferred to the coating [54,55]. This stress further increases with continued ion bombardment, and the structure densifies, which in turn drives the phase transformation from *h*-BN to *c*-BN and *w*-BN. The optimal bias voltage will introduce the stress required to convert soft *sp*^2^ bonded BN to the hard *sp*^3^ bonded cubic and wurtzite structure of BN.

Chen et al. [23] have discovered a stabilization mechanism for *w*-BN (produced utilizing the high pressure/high temperature approach) based on 3D networks of planar defects that are formed by dense network of intersecting stacking faults (ISF) and inversion domain boundaries (IDB). When the two orthogonal planar defects penetrate each other, an ISF–IDB junction is constructed to create a partial dislocation [23,56]. In GaN thin film, the empirical potential calculation model has shown that the ISF–IDB junction has low formation energy [56]. However, the *w*-BN to *h*-BN phase transition involves approximately ∼40% of lattice expansion of along the c axis. The conversion of *h*-BN from *w*-BN requires the movement of the partial dislocations at the ISF–IDB junctions along the c axis. This is energetically unfavorable and hence the ISF–IDB junctions in *w*-BN will suppress the transformation back to *h*-BN [23]. This mechanism contradicts the generally accepted opinion that crystal defects in materials will necessarily contribute to the initiation of phase transformations. In *h*-BN, N, and B sublattices are embedded together in the same c-plane whereas the two-neighboring c-planes are connected via weak van der Waals interactions. *h*-BN to *w*-BN phase transformation can be generated by applying a high compression stress to *h*-BN along the c-axis such that the N and B atoms at adjacent basal planes become close enough for direct chemical bonding. The N atom from a horizontal plane has an equal opportunity to bond with the upper and lower layer of B atoms. According to Chen et al., if the N atoms bond with the upper-layer B atoms on one side and bond with the lower-layer B atoms on the other side, an IDB is formed [23].

In our experiments, DC substrate bias of −400 V has converted *sp*^2^ bonded BN to hard *sp*^3^ bonded *c*-BN and *w*-BN. We suggest that the ion bombardment created by the substrate bias introduces compressive stress to facilitate *w*-BN formation. Stabilization of *w*-BN may occur via 3D networks of planar defects as described by Chen et al. but direct evidence for this is not yet obtained [23]. Our FTIR spectra bias-enhanced BN coating still shows a very weak hexagonal phase. X-ray photoelectron spectroscopy and X-ray diffraction pattern also shows a mixture of *sp*^2^ bonded BN and *sp*^3^ bonded BN. Although the bias-enhanced BN coating has both *sp*^2^ bonded BN and *sp*^3^ bonded BN, the hardness value of the coating increases by more than a factor of seven. Achieving high hardness with a mixture of *sp*^2^ and *sp*^3^ bonded BN is exciting; planned future experiments will involve varying the bias voltage in an attempt to fully convert *sp*^2^ to *sp*^3^ bonded BN. This should further increase the hardness of the coating to the superhard regime (>40 GPa). In order to better understand the mechanism of *sp*^3^ conversion and wurtzite stabilization, measurement of the coating stress, SEAD pattern by TEM and crystalline defects need to be obtained.

## 5. Conclusions

Boron nitride coatings were deposited in a microwave plasma chemical vapor deposition with and without DC bias applied to the substrate. The applied bias was found to introduce *sp*^3^ bonded boron nitride in both cubic and wurtzite structures, coexisting with the hexagonal phase. Formation of metastable wurtzite boron nitride using the low temperature plasma method is reported for the first time. This is in contrast to other reports that rely on high temperature–high pressure methods. Based on prior reports, it is expected that compressive stress is induced in the coating due to ion bombardment from substrate biasing. This stress can be responsible for converting *sp*^2^ to *sp*^3^ bonded boron nitride, but the observed stabilization of the wurtzite phase needs further study. Although the coating grown using applied bias has a mixture of *sp*^2^ and *sp*^3^ bonded BN, the presence of *sp*^3^ BN (in cubic and wurtzite forms) is enough to increase average nano-indentation hardness by more than a factor of seven compared to the coating grown without applied bias. Given the outstanding hardness of the wurtzite BN phase, even when compared to diamond, this represents an important finding with implications in achieving superhardness from mixed-phase BN materials.

## Figures and Tables

**Table 1:** Complete peak assignment for the (FTIR) spectral analysis of the boron nitride coatings.

Transmittance Frequency (cm^−1^)	Peak Assignment	References
786	Out-of-plane (B-N-B) bending modes of *h*-BN	[35,36]
1045	Restrahlen band of *c*-BN	[37,38]
1224	Transverse Optical (TO) mode of *w*-BN	[41]
1307	In-plane (B-N) stretching modes of *h*-BN	[35,36]

**Table 2:** XPS compositional analysis and fitted parameters of B1s and N1s.

Sample	Peaks	Binding Energy	Peak Area (%)	Assignment	References
Without Bias BN	B1s	189.8	100	*sp*^2^ BN	[44,45]
	N1s	397.2	100	*sp*^2^ BN	[44,45]
Bias-Enhanced BN	B1s	188.0	8	B–B	[34,46]
	B1s	190.5	62	*sp*^2^ BN	[42,45]
	B1s	191.8	30	*sp*^3^ BN	[42,45]
	N1s	398.2	65	*sp*^2^ BN	[42,45]
	N1s	399.5	35	*sp*^3^ BN	[42,45]

## Data Availability

The data presented in this study are available on request from the corresponding author.
